# Role of Telomeres Shortening in Atherogenesis: An Overview

**DOI:** 10.3390/cells10020395

**Published:** 2021-02-15

**Authors:** Yegor E. Yegorov, Anastasia V. Poznyak, Nikita G. Nikiforov, Antonina V. Starodubova, Alexander N. Orekhov

**Affiliations:** 1 Engelhardt Institute of Molecular Biology, Russian Academy of Sciences, Moscow 119991, Russia; yegorov58@gmail.com; 2 Institute for Atherosclerosis Research, Skolkovo Innovative Center, Moscow 121609, Russia; 3 Laboratory of Angiopathology, Institute of General Pathology and Pathophysiology, Moscow 125315, Russia; nikiforov.mipt@googlemail.com; 4 National Medical Research Center of Cardiology, Institute of Experimental Cardiology, Moscow 121552, Russia; 5 Institute of Gene Biology, Center of Collective Usage, Moscow 119334, Russia; 6 Federal Research Centre for Nutrition, Biotechnology and Food Safety, Moscow 109240, Russia; avs.ion@yandex.ru; 7 Pirogov Russian National Research Medical University, Moscow 117997, Russia; 8 Institute of Human Morphology, Moscow 117418, Russia

**Keywords:** atherosclerosis, telomeres, telomerase, LTL, cell senescence

## Abstract

It is known that the shortening of the telomeres leads to cell senescence, accompanied by acquiring of pro-inflammatory phenotype. The expression of telomerase can elongate telomeres and resist the onset of senescence. The initiation of atherosclerosis is believed to be associated with local senescence of the endothelial cells of the arteries in places with either low or multidirectional oscillatory wall shear stress. The process of regeneration of the artery surface that has begun does not lead to success for several reasons. Atherosclerotic plaques are formed, which, when developed, lead to fatal consequences, which are the leading causes of death in the modern world. The pronounced age dependence of the manifestations of atherosclerosis pushes scientists to try to link the development of atherosclerosis with telomere length. The study of the role of telomere shortening in atherosclerosis is mainly limited to measuring the telomeres of blood cells, and only in rare cases (surgery or post-mortem examination) are the telomeres of local cells available for measurement. The review discusses the basic issues of cellular aging and the interpretation of telomere measurement data in atherosclerosis, as well as the prospects for the prevention and possible treatment of atherosclerosis.

## 1. Telomeres

Cytologists call telomeres the end sections of chromosomes that are visible in a light microscope. Telomeres are large structures, covering areas of millions of DNA base pairs. Molecular biologists deal with a much smaller end region of the chromosome that overlaps thousands of nucleotide pairs. In the framework of telomere theory, telomeres are represented as short end regions, and it is this value that we consider in our review.

Telomeres are the end of a chromosome, so they have to be packed in such a way that repair systems do not recognize them as breaks. For this, natural selection has created special telomeric DNA sequences having the ability to fold in a special way and several proteins that cover these terminal structures. In humans and many other species, the telomeric repeat is represented by the sequence 5′-(TTAGGG) n-3′ [1].

Of particular interest in the structure of telomeres is their most distal part. The double strand of DNA does not reach the very end, the 5′-chain is shorter, and a single-stranded G-rich section remains. The length of the single-stranded 3′-end in humans is 100–150 nucleotides [2]. It is worth noting that the free 3′-end is available both in cells with functioning telomerase and without it. Its presence, therefore, cannot be explained by the activity of telomerase. 

The conformation of the single-stranded 3′-end attracts the attention of researchers, based on the thermodynamic features of a sequence containing repeating GGG clusters. Calculations show that such a sequence can easily form non-canonical structures (triplexes, quadruplexes). G-4 structures (quadruplexes) are formed by guanine tetrades. G-4 structures can be intramolecular, bimolecular, and tetramolecular. DNA strands inside G-4 structures can be parallel or antiparallel. It is believed that the formation of the G-4 structure requires a minimum of 12 guanins [3].

The experiments of De Lange and co. [4] led to the creation of a model of the telomeric structure, which is based on a DNA loop. According to this model, the free 3′-end, in complex with proteins, interacts with the double helix of telomeric DNA. The telomere thus forms a telomeric loop. According to the authors, the length of the telomeric loop correlates with the length of telomeres measured by independent methods. 

**Figure 1:**
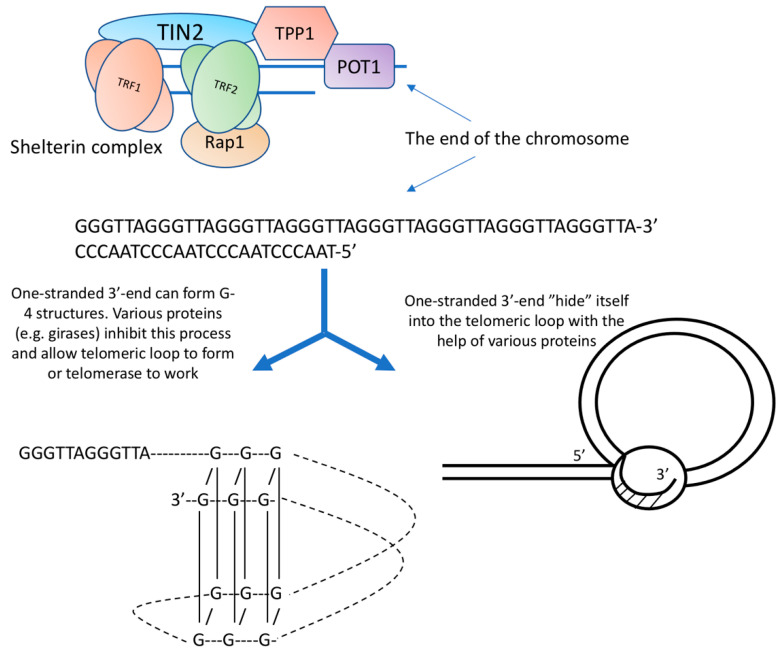
A highly simplified diagram of the molecular organization of telomeres.

Proteins that bind along the repeating path of telomeric DNA (TRF1 and TRF2 bind to double-stranded DNA regions, POT1 and TPP1 bind to single-stranded DNA regions) attach additional proteins (TIN2 and Pap1), which do not have, at least in humans, DNA-binding sites, and form a high-order nucleoprotein complex, which has been called shelterin. Refs. [5,6] (Figure 1).

## 2. Telomerase

Telomerase is a reverse transcriptase that uses a built-in RNA template to complete the end sequences of chromosome DNA. The basis of the enzyme is the protein part—human telomerase reverse transcriptase (hTERT) and the human telomerase RNA component (hTERC). There is a short region inside the RNA component of telomerase that serves as a template for telomeric DNA synthesis [7]. The enzyme works as a large complex of approximately 500 kDa including stably associated holoenzyme components (hTERT, hTERC, dyskerin complex, and TCAB1), and transiently associated proteins, such as pontin, reptin, and chaperones HSP90 and TRiC [8]. 

The hTERT gene consists of 16 exons and 15 introns. The total length of the hTERT gene is ~37 kb. p. In humans, 20 different variants of alternative splicing have been described [9]. There are known variants of splicing that strongly affect the level of telomerase activity [10]. Changes in splicing can occur not only during development, but also during carcinogenesis [11].

The hTERT promoter region is rich in sites for binding transcription factors. The presence of sites for numerous activators and repressors suggests a very complex system for regulating gene expression. The most well-known transcription factors that regulate hTERT transcription are c-Myc, estrogen receptor, HIF-1, NF-κB, Menin, STAT3, STAT5, MAD1, ETS, Sp1, Sp3, USF, NFX1, etc. [12].

Regulation of telomerase activity can occur at the stages of transcription, splicing, phosphorylation, maturation, and modifications of both hTERT and hTERC enzyme components [13]. The actual ability of telomerase to maintain telomere length also depends on several factors, including the localization of telomerase in the cell-nucleus or cytoplasm, the state of telomeric chromatin, changes in the packing of chromosome ends, etc. 

During the cell cycle, the intracellular localization of telomerase is regulated [14]. Before telomerase becomes able to work at the ends of chromosomes, it goes through a certain stage of maturation in Cajal bodies [15]. Cajal bodies contain a telomerase-specific protein, TCAB1 [16]. The actual suppression of telomerase can be achieved by delaying the enzyme in the nucleoli.

### Non-Canonical Telomerase Activities

Over time, information about some obscure functions of telomerase is accumulated that could not be explained by its role in maintaining telomeres or even by its mitochondrial functions. 

For example, increased mTERT (mouse) expression can accelerate skin wound healing and promote carcinogenesis without affecting telomere length [17]. Forced expression of telomerase can modify the inner functions of stem cells [18]. 

Telomerase has been shown to affect the activity of glycolytic genes [19] and independently of telomerase activity, it stimulates transcription of genes associated with the epithelial-mesenchymal transition (vimentin and snail1) [20]. Telomerase can regulate the work of NF-kappaB-dependent genes [21]. It has been shown that hTERT not only regulates the Wnt cascade but also vice versa [22].

Under conditions of oxidative stress, hTERT acts as a redox regulator, moving into mitochondria, where it protects mitochondrial DNA and maintains levels of anti-oxidative enzymes [23].

Forced expression of hTERT, via transcription factor STAT3, activates the expression of DNA methyltransferase I [24], participating in epigenetic regulation. 

In 2009, another surprising feature of telomerase was revealed, which allowed us to explain such a significant influence of telomerase on transcription [25]. It has been shown that the hTERT protein in complex with RMRP (the RNA component of mitochondrial RNA-processing endoribonuclease) has RNA-dependent RNA polymerase activity. hTERT-RMRP produces long double-stranded RNAs that are processed into 22-member siRNAs that specifically inhibit RMRP expression. RMRP mutations are associated with the cartilage hair hypoplasia (CHH) disease [26]. RMRP is essential for early development [27]. Possibly hTERT can form complexes with various RNAs and thus participate in the regulation of gene expression [28].

A rather interesting result was obtained after the introduction of the hTERT gene into the cells of a patient suffering from Newman–Pick disease. This is a type of inherited lipidosis in which the transport of cholesterol and glycosphingolipids from the lysosomes is disrupted. hTERT expression resulted in normalization of the cell phenotype [29].

## 3. Cell Senescence

### 3.1. How Does a Cell Measure the Length of Its Telomeres?

In 1997, two years before the discovery of telomeric loops [4], we proposed the hypothesis of the loop structure of telomeric DNA [30]. The hypothesis explains how cells measure their telomeres, why many carcinogenic viruses increase the proliferative potential of cells by 20–40 doubling, and why overexpression of telomere-binding proteins reduces the length of telomeres. 

**Figure 2:**
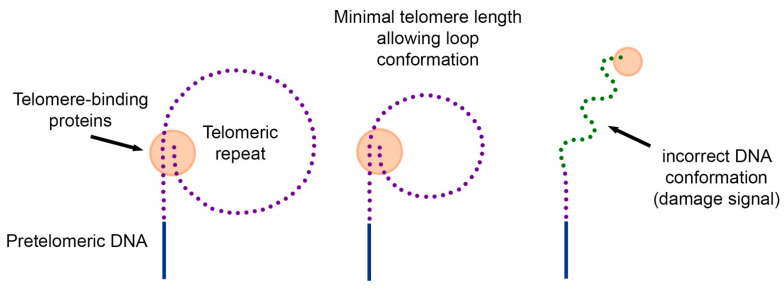
The hypothesis of the loop structure of telomeres. Telomeric DNA together with telomere-binding proteins can form loops. Due to the restriction of free rotation of DNA and interaction with various proteins, the DNA in the loop has a strained conformation. When telomeric DNA is shortened, there comes a point when the length of the telomeric repeat does not allow the loop to form. The telomeric end of the DNA acquires a free conformation, which is perceived by the cell as a signal of damage. As a result, the mechanism of replicative cell senescence is triggered.

Since DNA from the inner regions of chromosomes forms loops, and these loops can be used by the cell to quickly detect DNA breaks, we hypothesized that telomeric DNA can also form loops. The difference is in their size. If internal loops are hundreds of thousands of nucleotides long, then telomeric loops should have a minimum length of about several thousand nucleotides (Figure 2).

According to the proposed hypothesis, replicative aging occurs in cells when the length of the telomeric repeat decreases to such an extent that it becomes impossible to form a loop. Individual telomeres that do not form a loop become the source of the DNA damage signal. In this case, a change in intracellular signal transmission (lack of information about DNA tension) can restore proliferation. However, this proliferation will be limited to the length of a single loop.

### 3.2. DDR and SASP

Therefore, it is now established understanding that telomere shortening leads to the appearance of DDR (DNA damage response), which significantly changes many processes in the cell leading to the launch of the cell senescence program and the formation of the so-called SASP (senescence-associated secretory phenotype), characterized by pro-inflammatory changes [31]. 

The causes of telomere shortening can be, in addition to the end replication problem, damage to telomeres as a result of oxidative stress or DNA-damaging agents [32]. The senescence program can also be started when oncogenes are activated [33], when mitochondria are damaged (through oxidative stress) [34]. Regardless of the stressors, shortened telomeres fail to recover and DDR becomes permanent [32]. The result of constant DDR is activation of cyclin-dependent inhibitor pathways, including both the p53/p21Cip or p16Ink4a/Rb

Senescent cells of any origin gradually acquire SASP. SASP includes cytokines, chemokines, growth factors, proteases, and lipids. The transcription factors NF-κB, C/EBP, and p53 control the SASP [34].

It is believed that natural selection "invented" SASP for tumor suppression and wound healing. In an aging body, SASP causes chronic inflammation and contributes to the development of age-related diseases [35]. Removing senescent cells from the body significantly slows down aging and even promotes rejuvenation [36]. There are opinions that senolytics (the term originated in 2015) can cause significant improvements in the course of cardiovascular pathology [37].

## 4. Telomeres and Atherosclerosis

In developed countries, cardiovascular diseases (CVDs), mainly atherosclerosis and its consequences, are the leading cause of death in adults over 65 years of age. Although the first signs of atherosclerosis may appear as early as adolescence, the disease itself, which leads to severe complications, develops later. Since atherosclerosis has a distinct age dependence, it was clear from the very beginning that it is associated with aging. A striking example of age-related atherosclerosis is the Hutchinson–Guilford Progeria Syndrome (HGPS). Almost all of these patients die from a heart attack or stroke in their teenage years [38]. The underlying mutation of lamin A producing the abnormal protein, progerin, particularly affects tissues of mesodermal origin including the vascular system [39]. 

Although the mechanisms responsible for the link between progerin expression and telomere shortening remain poorly understood, HGPS patient-derived fibroblasts reach senescence in culture prematurely and their telomeres shorten faster [40]. In this case, ectopic expression of telomerase suppresses the proliferative defect [41]. In addition to influencing proliferation, telomerase expression reverts progerin-induced changes in gene expression, and these changes are in many ways linked to senescence [42]. All this indicates that progerin induces telomere dysfunction, and telomerase is capable of protecting cells from the toxic effects of progerin. 

### 4.1. Atherosclerosis Is Associated with the Cell Senescence

After Hayflick’s work, when cytogerontology emerged, the pathogenesis of atherosclerosis has been associated with cell senescence. A significant shortening of telomeres in endothelial cells in the areas of atherosclerotic lesions was shown, and the appearance of markers of aging (senescence-associated beta-galactosidase) was demonstrated. Morphological changes in the endothelium also indicate its aging: disturbed cell shape, increased size, accumulation of material in the cytoplasm [43,44,45,46]. The reasons causing the senescence of endothelial cells are seen in their constant reactions to mechanical stimulation by the current of fluid. The location of atherosclerotic plaques is clearly associated with blood flow hydrodynamics. Many studies have shown that both initiation and progression of plaques are associated with either low or multidirectional wall shear stress (WSS) [47,48]. Low or oscillatory WSS increases ROS and NO production and enhances NADPH and xanthine oxidase expression and eNOS genes expression in endothelial cells, which lead to the induction of oxidative stress [49]. It induces expression of VCAM-1 and ICAM-1, production of MMPs resulting in ECM degradation. Additionally, after all, low WSS can stimulate vascular smooth muscle cell migration from the media to the intima, followed by proliferation that results in the formation of a fibrous cap. At the sites of low or oscillatory WSS, mitotic and apoptotic endothelial cells are observed [50]. By contrast, laminar WSS produces anti-inflammatory effects, and endotheliocytes are in the quiescent state.

Despite the very great efforts aimed at studying atherosclerosis, there are still different theories of its development and humanity spends huge efforts on atherosclerosis treatment and prevention.

In this regard, research has been conducted and is being conducted on the relationship of atherosclerosis with aging. The telomeric theory of aging is now generally accepted; additionally, telomere measurements are a fairly clear quantitative feature, so they have been widely developed. Research on atherosclerosis in humans is extremely difficult, because studying the vessel material (where the disease is developing) is possible only on postmortem materials or biopsies obtained during surgical operations. It is impossible to take vascular biopsies and look at the length of telomeres in them. Therefore, all we are given is almost a search under a lantern, to take biopsies from available places (from blood cells). This causes many difficulties in interpreting the data.

### 4.2. Various Factors Affect the Length of Telomeres 

Despite these difficulties, numerous studies have shown that the average length of telomeres in the blood leukocytes (LTL) of patients with atherosclerosis is shortened and corresponds to the length of people 11 years older [51]. LTL shortening also occurs in young patients with ischemic heart disease [52].

In recent years, a meta-analysis of numerous publications on the relationship of LTL with the risk of atherosclerosis development and its complications, including strokes, heart attacks, and heart failure, has become widespread [53,54]. A meta-analysis of coronary disease (including 18 publications) proved that LTL shortening is very significant for the risk of developing angina pectoris [55,56]. Accumulated evidence shows that short LTL is not only associated with stroke occurrence, but also associated with post-stroke recovery in the elderly population [57].

However, data does not always have logical explanations: LTL was not identified as a marker of acute myocardial infarction and also as a predictor at medium-term follow-up in the study of a cohort of young Italian patients [58].

In contrast to expectation, LTL was positively associated with left ventricular mass and wall thickness in patients with hypertension [59] and in normotensive subjects [60]. Changes in LTL may be associated not only with cardiovascular reasons, but also with cerebrovascular disease manifestations [61].

During a 9.5-year follow-up period, enhanced telomere shortening was not associated with carotid atherosclerosis. Short telomere length was more strongly associated with early-onset than late-onset carotid atherosclerosis. The results of this study demonstrated that the enhanced telomere shortening during adult life possibly does not explain the short telomeres observed in subjects with atherosclerotic disease. It is more likely that the clinical manifestation of atherosclerosis is predicted by pre-existing telomere shortening [62].

Unusual results were obtained when studying LTL in smokers: smokers have shorter telomeres, but the rate of their shortening is equal to the control. The authors conclude that the most likely explanation is that people with shorter telomeres start smoking [63].

To explain the revealed discrepancies between telomere length in leukocytes and manifestations of several diseases, it should be noted that, first, LTL is not a very specific marker of atherosclerosis. Experimental data further reveal that hypertrophic hearts with reduced ejection fraction exhibit the shortest telomeres [64]. Shortened leukocyte telomere length demonstrates a significant association with stroke, myocardial infarction, and type 2 diabetes mellitus. A short leucocyte telomere length is associated with the development of insulin resistance [65]. Larger, well-designed studies are needed to explore sources of heterogeneity [66].

LTL is most strongly associated with adiposity, but is also associated with biomarkers across several physiological systems. LTL may thus be a predictor of cardiovascular disease through its association with multiple risk factors that are physiologically correlated with risk for the development of cardiovascular disease [67].

Variability is the second (after the low specificity) problem. Significant telomere length variation exists between individuals, which begins at birth. This variation is ≈ 3 times larger than the variation in telomeres length (TL) within the somatic tissues of individuals [68].

The third problem is that age-related changes in telomere length can mask changes associated with atherosclerosis. The age dependence of the LTL length is well known. But it should be noted that the main (defining) telomere shortening occurs in early childhood and changes are relatively small after the age of 10. Thus, (defining) telomere shortening occurs in early childhood and changes are relatively small; heritability and events in early childhood are the main factors that determine the length of telomeres throughout life [69,70].

Some authors express extreme judgments that changes in adult lifestyle have little effect on telomere length [71].

A meta-analysis on a large sample indicates that telomere length is a well-inherited parameter that is influenced by strong maternal heritability and the age of the father at conception [72]. Strong maternal inheritance indicates the possible involvement of mitochondria in determining the dynamics of LTL, which complicates the analysis in the case of atherosclerosis due to the participation of macrophages in the pathogenesis. In recent years, it has become clear that mitochondria determine a significant proportion of the pathogenesis of atherosclerosis. There are mitochondrial haplogroups that are resistant to atherosclerosis; with aging, mitochondrial mutations accumulate, which increases the severity of atherosclerosis. In general, telomere shortening largely depends on the state of the mitochondria. Additionally, mitochondrial defects lead to increased inflammation, including an increase in the inflammatory phenotype of macrophages. Unfortunately, due to the limited volume, this topic cannot be adequately covered in our review [73,74,75]. 

Inter-tissue differences in telomere length, as well as the telomere length itself, are also established at an early age, after which a stable correlation between different tissues is observed. Low-proliferating tissues (muscle, fat) have the longest telomeres, while proliferating tissues (blood, skin) have shorter telomeres [76].

At the same time, the length of telomeres and their age dynamics differ in lymphocytes and granulocytes [77]. It seems that these differences indicate the history of the diseases of the particular individual. This means that when performing mass measurements of blood cell telomeres to find a link to chronic diseases, it would be highly desirable (or even necessary) to perform separate measurements of granulocytes, lymphocytes, and monocytes. Such measurements can serve as one of the options for internal control.

An even more advanced control can be parallel measurements of telomere length in any non-dividing tissues. In some studies, when more than just blood cells become available, internal controls can be used to eliminate inter-individual differences. The authors of the study cardiac surgeons found that in the muscle cells of the right atrium, telomere shortening occurs to a minimum extent (up to 60 years of telomere length practically does not change), and the difference between LTL and the length of the right atrium telomeres becomes a more significant parameter than LTL, and this difference determines the prognosis of treatment [78].

We can provide one more additional consideration regarding LTL and atherosclerosis. Attention should be paid to the following circumstance.

LTL is a parameter that extends to the entire body, but atherosclerosis is a fairly local (limited) phenomenon. Therefore, LTL (changes in blood cells) should definitely and strongly affect the course of atherosclerosis, while atherosclerosis, especially in the early stages, should occur quite imperceptibly for cells from the bone marrow (atherosclerosis is not diagnosed using the blood formula).

The state of carriage of somatic mutations of blood cells—CHIP (clonal hematopoiesis of indeterminate potential) is characterized by a 2–4-fold increase in the risk of developing cardiovascular diseases. The reasons for this are not clear. At the age of 70, more than 10% of individuals are carriers of mutant cell clones, which at the same time make up about 20% of leukocytes [79].

Short telomeres are known to increase genetic instability and promote mutation and, in the case of blood cells, myelodysplastic syndromes [80]. One possible explanation for the CHIP phenomenon is that short telomeres can contribute to the development of CHIP and independently increase the likelihood of developing cardiovascular pathology. However, this explanation is contradicted by the fact that experimental modeling of CHIP in mice reveals a pro-inflammatory phenotype of macrophages [81].

In Table 1, we summarized data on methods used in the measurements of telomeres in studies we mentioned in this section.

## 5. Pathogenetic Features of the Mechanisms Involved in the Development of Local Inflammation in Atherosclerosis

It is logical to consider atherosclerosis as a disease associated with the local aging of endothelial cells, as stated in the 1990s by Calvin Harley (personal communication). However, in fact, atherosclerosis is more than cell senescence. Initially, the senescence of the endothelium attracts cells of the immune system (senescent cells are removed from the body with the help of immune cells), and then, for several reasons, an inflammatory process occurs that does not have a resolution, and it is this inflammatory process (quite local for the body) that has fatal consequences.

**Figure 3:**
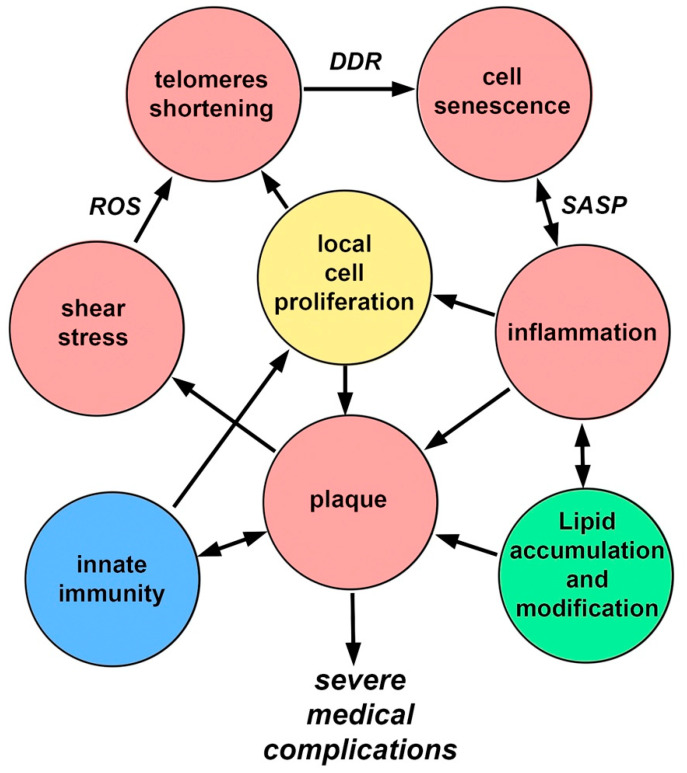
Positive feedbacks in the pathogenesis of atherosclerosis.

In the pathogenesis of atherosclerosis, positive feedbacks (vicious circles) are clearly visible, which prevent the completion of the process (Figure 3). These connections have pronounced temporal dynamics, which emphasizes the connection with the body aging again. Endothelial cell senescence, induced by the features of WSS, causes the appearance and development of a bulge on the vascular wall (plaque), which in turn increases flow turbulence and contributes to the further development of cell senescence. Over time, artery stiffness increases that contributes to increases in arterial blood pressure, and induces greater oscillatory WSS [82].

Endothelial damage leads to the development of inflammation, which leads to the appearance of SASP, which, in turn, promotes the development of inflammation involving monocytes. Monocytes, in addition to their presence, stimulate the proliferation of smooth muscle cells. The plaque grows due to the migration of monocytes, the proliferation of smooth muscle cells, and the ongoing accumulation of lipids. All together, these processes increase the effect of WSS. Inflammatory changes promote lipid adsorption. Lipids in the plaque are gradually oxidized and become pro-inflammatory, which enhances the recruitment of monocytes, etc. [83,84].

Aging by itself, even without the development of atherosclerosis, increases the presence of pro-inflammatory changes in the arterial wall [85]. Inflammatory signaling stimulates ROS production (and vice versa) through various mechanisms [86]. A relatively hypoxic microenvironment is created in the plaque, which contributes to the transition of energy supply to glycolysis, which, in turn, possibly contributes to the metabolic reprogramming of macrophages along the classical (inflammatory) pathway, which contributes to inflammation [87]. Aging and chronic inflammation can damage mitochondria, which then contribute further to the process, either by releasing mitochondrial DNA into the cytoplasm or by increasing ROS production. Energy metabolism may shift towards glycolysis, which leads to metabolic reprogramming of macrophages. The emergence of mitochondrial DNA triggers innate immunity mechanisms [88,89,90]. Finally, with age, the number of mitochondrial mutations in blood cells increases, which enhances the above mechanisms due to migrating monocytes [91].

Therefore, the development and prognosis of atherosclerosis are determined by two, related but different processes: cell senescence and inflammation. Their incomplete concordance may complicate the relationship between the length of telomeres in blood cells and the prognosis of atherosclerosis.

Both telomere length and immune system features have individual differences, and both of these traits change with aging. In addition to shortening telomeres, aging causes somatic mutations and mutations in the mitochondrial genome. All these processes usually aggravate the course of atherosclerosis.

From a practical point of view, the most important questions are the behavior of already formed atherosclerotic plaques and their stability or tendency to rupture and form a blood clot [92]. Probably, these processes are very strongly linked to the mechanisms of inflammation.

Some success in the fight against cardiovascular diseases (mainly atherosclerosis) can be achieved by giving up bad habits, choosing a diet [93], and maintaining moderate physical activity. These exposures, called proper lifestyles, slow down aging, reduce inflammation [94], and reduce the number of calories consumed and blood-tissue transport of lipids. It is quite logical that this complex works. However, this is not a solution to the problem of atherosclerosis. In the modern world, there is an increase in the average age, a decrease in physical activity, the transition of life to an online format, and in developed countries, the availability of food is increasing. The development of methods of active influence on atherosclerosis is required.

## 6. Consider Targeting Telomeres and Telomerase

If we approach the treatment of atherosclerosis from the standpoint of affecting telomeres, telomerase, and senescence, several options can be assumed. One of them is an increase in telomerase activity to lengthen telomeres.

There are proposals for the treatment of atherosclerosis by affecting telomeres/telomerase [95,96,97]. Sequence variation in telomerase reverse transcriptase (hTERT) is a determinant of the risk of cardiovascular disease: the Atherosclerosis Risk in Communities (ARIC) study [97].

When proposing a therapeutic approach associated with telomerase activation, it is necessary to discuss safety issues, primarily the dangers of induced carcinogenesis. First of all, it should be noted that activation of telomerase in normal cells does not lead to any changes associated with carcinogenesis, except for the possibility of endless growth, which in itself does not lead to carcinogenesis [98,99]. Many normal (not tumorigenic) cells have telomerase activity. They are most cells in a developing fetus, normal stem or progenitor cells in highly proliferative compartments. The male germ line tissue in testes has high constitutively active telomerase. Trying to highlight the safety of telomerase, Calvin Harley even published an article titled: “Telomerase is not an oncogene” [100].

On the other hand, telomerase activation is the most common feature of cancer cells (about 90%), and in this regard, it is one of the main markers of cancer [101,102]. One can imagine the following situation. For the formation of a cancer cell, a certain set of changes is required, one of which is associated with immortalization. If a number of changes have already taken place (the cell has become precancerous), then the addition of active telomerase will promote the formation of full-fledged cancer cells. The probability of such a scenario is generally still unknown, but it is impossible to exclude it. However, it should be noted that cancer growth requires a constantly working telomerase with high activity. It is known that tumor malignancy correlates well with the level of telomerase activity [103,104]. Moreover, the level of telomerase activity can differ hundreds of times [105]. Based on these arguments, it can be assumed that the induction of high, unregulated telomerase activity in the cells of aging of the organism, which has managed to accumulate a significant number of mutations, contains a well-defined threat of increased carcinogenesis. However, a relatively low and, possibly, intermittent telomerase activity should allow cells only to “heal” telomeres, thereby decreasing DDR and SASP and reducing inflammation, but not ensuring long-term growth [106]. The possible protective effect of telomerase under conditions of oxidative stress cannot be disregarded as well [23]. In any case, the question of the application of telomerase for the treatment of pathologies associated with aging needs to be further studied [107].

In our hands, we have twice observed the appearance of chromosomal aberrations after long-term (over 300 population doubling) cultivation of human cells with the introduced hTERT gene (unpublished results). The process of change took several years: this is a lot for a scientific experiment, but how much is it for human life? In any case, modern systems of introducing genes with their subsequent integration carry increased risks that are incompatible with the possible positive effect on diseases associated with aging.

Gene therapy using adeno-associated viruses is relatively safe, because it does not lead to an integration of the construct into the genome. By introducing the telomerase gene into aging mice, significant results have been achieved in improving biomarkers associated with aging [108].

Unfortunately, the authors did not investigate the issue of atherosclerosis.

A similar result in mice was achieved by the same authors using a low-molecular-weight telomerase activator TA-65 instead of AAV9 [109]. 

TA-65 is a natural product derived from a Traditional Chinese Medicinal plant (Astragalus). Extracts of this plant have been used for centuries without adverse effects reports. The dosage of the drug is easy to control and is quite a weak telomerase activator [109]. Recently, a randomized, double-blind study was conducted that showed that TA-65 improved key markers of cardiovascular disease risk. Patients received either 16 mg daily of a TA-65 supplement or a placebo for 12 weeks. LTL did not change significantly over time. However, the study found that subjects had lower body mass index and waist circumference after the supplement period (*p* < 0.05). The LDL-C/HDL-C ratio (*p* < 0.05), a key marker of cardiovascular disease risk, decreased. The level of TNF-α was also reduced. The authors conclude that there have been changes associated with reductions in inflammation [110]. 

Telomerase Activator to Reverse Immunosenescence in Acute Coronary Syndrome: A Double-Blind, Phase II, Randomised Controlled Trial investigating the effect of the small molecule activator TA-65, which can potentially restore telomerase activity and prevent T cell senescence, is currently underway (Phase II) [111].

It is known that Telocyte (www.telocyte.com (accessed on 26 January 2021)) (a preclinical gene therapy company) intends to initiate trials using an AAV9 vector and hTERT to reset cell aging in vivo.

Temporary activation of telomerase is possible in different ways, and recovery of telomerase activity in somatic cells may have therapeutic effects. This was shown to recover the telomere length and thus to reverse cell senescence and restore a functional phenotype [112]. A correlation was observed between telomere attrition in cells of the cardiovascular system and the development of atherosclerosis in animal models and humans [113]. Moreover, shortened telomere length of circulating lymphocytes, used as an indirect marker of a declining pool of circulating progenitors, has been identified as a predictor for the early onset of cardiovascular disease [114].

Results of preclinical studies suggest the significant potential of telomerase activation to delay or even reverse the senescent phenotype of aged vascular cells [115].

It is possible to use therapeutic RNA to transiently express proteins of interest for a defined period [116]. The effect of modified mRNA that encodes telomerase was estimated in senescent human cells, and the telomere length and ability to replicate were found to be increased [117]. However, this would require a method for delivering the therapeutic RNA to the vasculature. RNases have a ubiquitous nature, which makes the half-life of therapeutic RNA very short without some protection. Moreover, the systematical administration of RNA can cause an activation of innate immunity and lead to severe adverse effects development.

To solve this problem, RNA modifications, for example, modified nucleotides that reduce innate immune activation, and also novel delivery devices can be used. Inflammatory markers ICAM-1 and VCAM-1, which are differentially expressed on the surface of the senescent endothelial cells, offer a potential target for enhancing delivery. Functionalized nano- and microparticles were shown by Tasciotti’s group to be able to release therapeutic molecules to sites of inflamed endothelium [118]. Experimental results suggest that anti-inflammatory drugs and other therapeutic compounds can be packaged into nanoparticles that are partially composed of autologous leucocyte membranes. The interaction of the leucocyte surface markers with the inflammatory surface markers on the inflamed endothelium allows the region-specific release of the therapeutic agent. Targeting the endothelium using nanoparticles that preferentially deliver RNA encoding telomerase is one such strategy. Endothelial regeneration would be expected to slow or reverse vascular diseases and may provide a higher quality of life to the aging population [95].

Chronic removal of aging cells in the experimental model improves the condition of atherosclerosis [40]. Zhu and colleagues have shown the potential improvement of the myocardial function of aged mice in response to pharmacological elimination of senescent cells. 24-month old mice were treated with a single dose of D&Q (dasatinib and quercetin). This significantly improved left ventricular ejection fraction and fractional shortening. Such a change in function was suggested to be a result of a restoration in vascular endothelial function, but no change in smooth muscle contractile function was observed [39]. 

However, this study does not provide the quantitative data of myocardial senescence before or following senolytics treatment. It is still not clear whether an accumulation of senescence in the endothelial cell population or paracrine signaling via the SASP from other senescent cell populations underlies endothelial cell dysfunction.

The study by Childs et al. revealed that the treatment of p16-INKATTAC, P16-3MR, and P16-NTR transgenic mice with the navitoclax can inhibit atherogenesis, which is indicated by the reduction in plaque number and plaque burden and the average size of individual plaques [37]. The beneficial effect of navitoclax was confirmed by the use of genetic mouse models with the eliminated senescence. The same result was obtained by the D&Q senolytic treatment in an alternative atherogenesis model, ApoE^−/−^ mice, which develop atherosclerotic plaques containing increased numbers of senescent cells on a high-fat diet. D&Q treatment reduced senescence burden and plaque calcification, although no difference in plaque size was observed [119]. Navitoclax treatment was also shown to rescue immunosenescence. It is supported by the decrease in CD8+ effector memory cells and an increase of the naïve CD8+ T cell population [120]. The mechanism underlying this is unclear, but the senolytic treatment potentially influenced the dynamics of lymphocyte proliferation, as a result of reduced systemic inflammation or rejuvenation of progenitor pools, which in turn changed the balance of different T cell subpopulations [121].

An interesting approach with suppression of the aging phenotype in a transgenic HGPS mouse model has been demonstrated recently. The introduction of sequence-specific telomeric antisense oligonucleotides (tASOs) prevented full DDR development during telomere shortening and significantly enhances skin homeostasis and lifespan [122]. This work actually presents an alternative to the use of senolytics: instead of removing senescent cells, it is possible to suppress the processes leading to the development of SASP. 

Recently, it was found that repeated intermittent hyperbaric oxygenation is able to affect cells of the immune system (T-helper, T-cytotoxic, natural killer, and B-cells), leading to significant lengthening of telomeres and a decrease in the number of senescent cells [123]. During such an impact, the organism is trained to hypoxia; a hyperoxic–hypoxic paradox occurs [124], which can enhance mitochondrial biogenesis and cause the mobilization of stem cells [125]. An improvement in oxidative metabolism to promote lipid consumption has to occur [126]. On the one hand, this effect is senolytic (decreasing SASP), on the other hand, this effect can be assessed as a rejuvenation of the immune system. It is possible that this effect alters the pro-inflammatory/anti-inflammatory balance and contributes to the resolution of inflammation [126], because hyperbaric oxygen therapy is a well-established treatment modality for non-healing wounds. It would be extremely interesting to evaluate the effect of hyperbaric oxygenation on the course of atherosclerosis.

## 7. Conclusions

Atherosclerosis is a disease with a very long development. Atherosclerotic changes are detected at a fairly early age, but the clinical consequences occur much later. Natural selection has put a significant margin of safety in the human design; men usually go to the doctor with heart problems when the cross-sectional area of the coronary vessel is reduced many times due to the development of thickening. 

Aging is present in the pathogenesis of plaque, but the development of a particular plaque also depends on the level of inflammation and lipid metabolism. Capillary endothelial cells are involved in the transport of lipids from the bloodstream to the organs. It is possible that endothelial cells in the area of atherosclerotic lesions act as the endothelium of capillaries, trying to transport lipids to the organ, but instead, the lipids are trapped in the arterial wall. Accumulation and long-term storage of lipids leads to their oxidation, the appearance of oxylipids that contribute to inflammation.

Lipids are poorly soluble in water, and when their carriers are not enough, they dissolve in the membranes, disrupting their functions. The body saves on lipids. When they are eaten, they are not completely broken down, and their blocks (fatty acids) are used readily. Therefore, in the case of lipids, the expression we are what we eat takes on a direct meaning. The toxicity of lipids leads to the fact that the cell tries to isolate them and sends them to the lysosomes, and the process begins to drag on.

At a more mature age, the specific harm from atherosclerosis begins to depend on the stability of the plaque and on the intensity of the local inflammatory process, which contributes to vessel obstruction and can also lead to plaque destruction and embolic process. The inflammatory process involves cells of the immune system that come from the bone marrow. They are also susceptible to aging, as are local cells. In addition to shortening telomeres in cells of the immune system, mitochondrial DNA mutations accumulate in them with age, which contributes to the instability of mitochondria, which leads to their destruction and activation of innate immunity systems, which results in a pro-inflammatory phenotype of macrophages arriving at the site of damage to the vascular wall.

Mitochondrial dysfunction can occur without the involvement of the mitochondrial DNA alterations. In this case, the mitochondria begin to produce an excess of ROS, which leads to accelerated shortening of telomeres. The protein component of telomerase can respond to oxidative stress and reduce its destructive effects. In addition, telomerase activators can act at other levels, reducing the intensity of the inflammatory process, first of all, locally, reducing DDR signaling in the endothelium and smooth muscle cells, as well as systemically, affecting immune cells. Further research is required to establish the connection between the aging of local cells (endothelium and smooth muscle cells), cells of the immune system, with the processes of inflammation and lipid accumulation in the area of atherosclerotic damage.

## Figures and Tables

**Table 1:** Details of telomeres measurements.

Method	Sample Size	Reference
Terminal restriction fragment using Southern analysis	51 subjects (23 males and 28 females, 37 White Americans, 14 African Americans) between ages of 1 month and 80 years.	[43]
Terminal restriction fragment using Southern analysis	11 patients with CAD and 22 patients without CAD	[44]
Terminal restriction fragment using Southern analysis	13 patients	[45]
Terminal restriction fragment using Southern analysis	203 cases with a premature MI (50 years) and 180 controls	[51]
Modified monochrome multiplex quantitative PCR method	105,055 individuals from Copenhagen (17,235 were diagnosed with ischemic heart disease between 1977 and 2013) Coronary ARtery DIsease Genome wide Replication and Meta-analysis (CARDIoGRAM) consortium dataset (184,967 participants, 60,837 cases of ischemic heart disease)	[52]
Modified monochrome multiplex quantitative polymerase chain reaction method	19,838 Danish general population participants	[53]
qPCR	1511 CAD patients; 1553 control	[56]
quantitative PCR-based method	199 patients from 18 to 48 years old with first diagnosis of acute myocardial infarction; 190 control	[58]
Southern blot analysis	850 Framingham Heart Study participants (mean age 58 years, 58% women)	[59]
quantitative polymerase chain reaction	334 randomly selected Flemish participants (mean age 1⁄4 46.5 years; 52.5% women)	[60]
quantitative polymerase chain reaction (qPCR)-based assay	2211 healthy individuals and 2140 CHD patients	[61]
Southern blots	154 French men and women (aged 31–76 years at baseline)	[62]
Q-FISH	63 samples	[64]
Southern blots	756 intact twins pairs	[65]
quantitative PCR	9191 participants aged 20–84	[67]
Southern blots of the terminal restriction fragments (TRFs)	1156 adult (44% women)	[71]
Terminal restriction fragment using Southern analysis	87 adults (aged 19–77 years)	[76]
FISH	835 healthy individuals and 60 individuals with reduced telomerase activity	[77]
qPCR	163 patients who underwent cardiac surgery	[78]
quantitative fluorescence in situ hybridization	58 patients with myelodysplastic syndrome	[79]
